# Reversible severe mitral regurgitation during immunotherapy in a structurally vulnerable valve: a case report

**DOI:** 10.1093/ehjcr/ytag474

**Published:** 2026-06-26

**Authors:** Sachin Singh, Ushma Majmudar, Indu Poornima, Anita Radhakrishnan

**Affiliations:** Department of Internal Medicine, Central Michigan University College of Medicine, 1000 Houghton Ave, Saginaw, MI 48602 USA; Department of Cardiovascular Medicine, Allegheny Health Network, 320 E North Avenue, Pittsburgh, PA 15212, USA; Department of Cardiovascular Medicine, Allegheny Health Network, 320 E North Avenue, Pittsburgh, PA 15212, USA; Department of Cardiovascular Medicine, Allegheny Health Network, 320 E North Avenue, Pittsburgh, PA 15212, USA

**Keywords:** Mitral regurgitation, Blinatumomab, Fenfluramine, Cardio-oncology, Drug-induced valvulopathy, Case report

## Abstract

**Background:**

T-cell therapy (TCT) rarely causes isolated severe valvular pathology without cardiomyopathy, but its haemodynamic effects can exacerbate mitral regurgitation (MR) in patients with subtle valvular issues, and reports describing reversible valvular dysfunction in this context remain limited.

**Case:**

A 48-year-old woman with a history of fenfluramine (FF) use for weight loss 10 years prior was diagnosed with B-cell acute lymphoblastic leukaemia (B-ALL). Baseline echocardiogram revealed trace MR. After her second blinatumomab (BL) cycle, she developed acute heart failure symptoms. Repeat echocardiogram showed severe MR with posterior leaflet restriction. Systemic inflammatory symptoms and end-organ damage were notably absent. BL was held, and medical therapy for heart failure with preserved ejection fraction (HFpEF) was initiated, improving MR and allowing resumption of BL.

**Decision-making:**

Baseline posterior mitral leaflet restriction was likely related to prior FF use. While BL carries a low cardiotoxicity profile, it can cause heart failure via cytokine release syndrome (CRS)-dependent and potentially CRS-independent pathways. In this case, we hypothesize that transient elevations in left-sided filling pressures, possibly from unmasking subtle diastolic dysfunction in a structurally vulnerable valve, amplified her MR. Delayed disclosure of FF use underscores the importance of intentional, non-judgmental history-taking. Recognition of this interaction guided haemodynamic optimization and enabled cautious, successful reinitiation of BL therapy.

**Conclusion:**

We emphasize considering prior FF use when evaluating patients with idiopathic MR. We also demonstrate how haemodynamic changes from TCT can worsen MR and show how careful haemodynamic management can enable the safe resumption of this lifesaving therapy.

Learning points

- Severe mitral regurgitation during immunotherapy may reflect haemodynamic stress on a structurally vulnerable valve rather than irreversible drug-related cardiotoxicity.
- In patients with remote fenfluramine exposure or subtle valvular abnormalities, prompt haemodynamic optimization can allow safe continuation of immunotherapy.
- Temporary immunotherapy interruption, rather than permanent discontinuation, should be considered when valvular dysfunction is haemodynamically driven and reversible.

## Introduction

Blinatumomab (BL), a bispecific T-cell engager (BiTE) that binds CD3 on T-cells and CD19 on malignant B-cells, improves event-free and overall survival in adults and children with B-ALL.^1^ A 2024 pharmacovigilance study reported cardiovascular adverse events in 20.4% of BiTE recipients.^2^ These events include arrhythmias, hypotension, and tachycardia and are often observed in the context of cytokine release syndrome (CRS), which occurs in ∼14% of patients, with severe (grade ≥3) CRS is reported in 7% of cases.^3^ CRS itself is driven by an interleukin-6 surge that raises systemic vascular permeability, myocardial oxygen demand, and left-sided filling pressures.^4,5^ Cardiovascular events, including heart failure, may also occur independently of overt CRS, and their haemodynamic impact in patients with structurally abnormal valves remains poorly characterized.

Drug-induced valvular heart disease remains clinically relevant more than two decades after the withdrawal of fenfluramine–phentermine. Histopathology reveals serotonin-mediated fibroelastic thickening of leaflets and subvalvular apparatus, classically affecting the right-sided valves but with up to 23% cases involving the mitral valve.^6–8^ Importantly, leaflet restriction can persist long after drug cessation, potentially rendering the valve susceptible to haemodynamic stressors.^9^

These considerations underscore the importance of early cardiovascular risk stratification in patients receiving immunotherapy.^10^ In this context, we describe a case of BL-related, CRS-independent heart failure that transiently converted trivial MR to severe MR and highlight the role of guideline-directed medical therapy (GDMT) in such patients.

## Summary figure

Progression from trivial to severe mitral regurgitation during blinatumomab therapy, followed by improvement with temporary treatment interruption and guideline-directed medical therapy, allowing safe resumption of immunotherapy.

Abbreviations: MR = mitral regurgitation; TTE = transthoracic echocardiogram; ED = emergency department; LVEF = left ventricular ejection fraction; SGLT2i = sodium-glucose cotransporter-2 inhibitor; ARB = angiotensin receptor blocker; HFpEF = heart failure with preserved ejection fraction; HF = heart failure.

**Figure:**
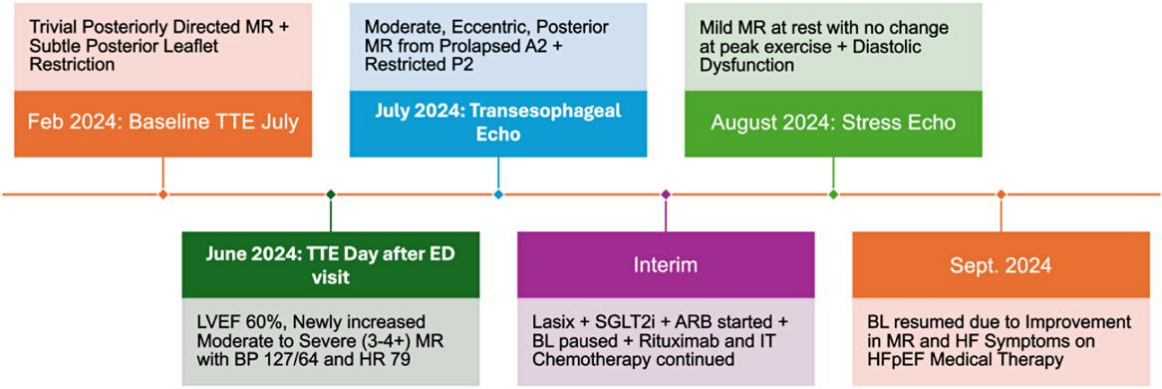
Timeline of mitral regurgitation severity and management

## Case

A 48-year-old woman with a history of hypertension, fibromyalgia, migraines, depression, and obesity (body mass index of 31 kg/m^2^), with prior weight-loss drug exposure, presented to the Cardio-Oncology clinic with worsening dyspnoea and ankle oedema.

**Figure 1:**
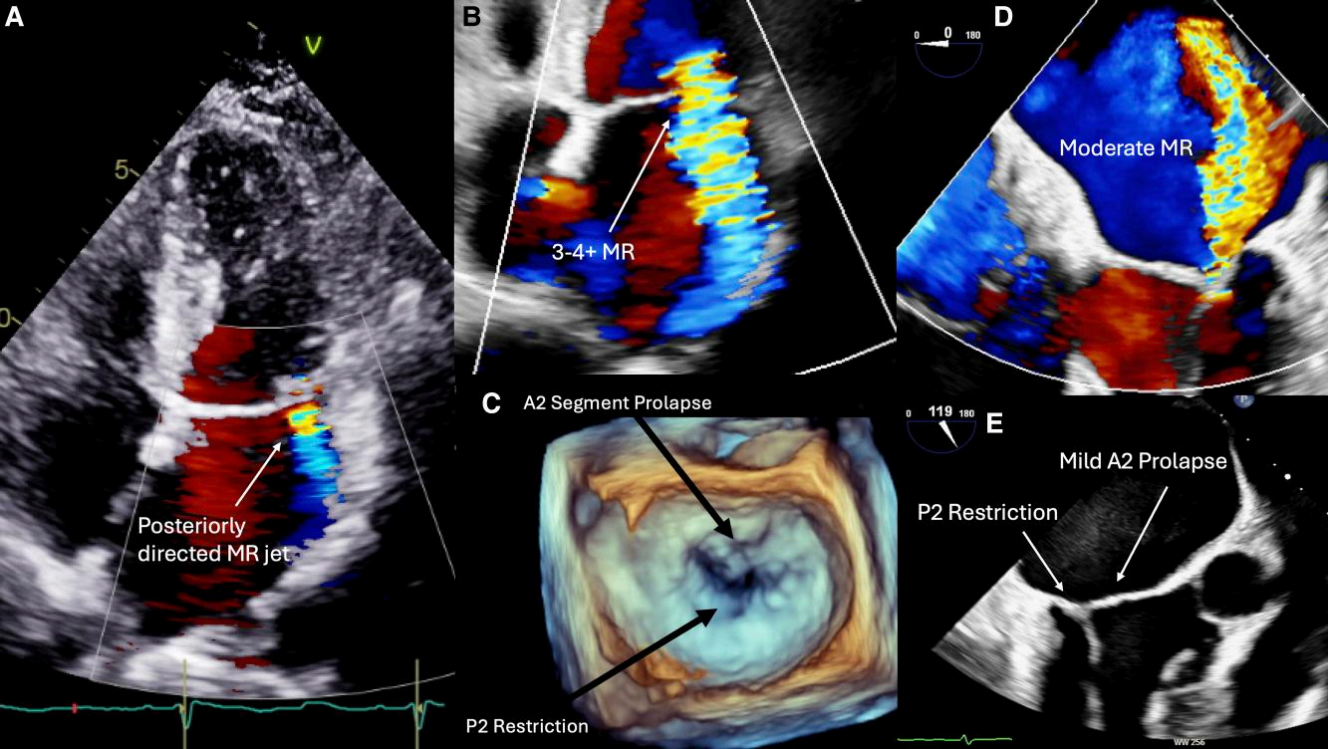
Echocardiographic images at various time points. (*A*) Baseline TTE in February 2024 showing posteriorly directed trivial MR in the Apical 4-chamber view. (*B*) Moderate to severe MR noted on July 2024 TTE in the apical 4-chamber view (*C*) 3D surgeon’s view of mitral valve with pseudo-prolapse of A2 segment due to significantly restricted P2 segment on TEE (*D*) Moderate posteriorly directed MR at 0° mid-oesophageal view on TEE. (*E*) 120° mid-oesophageal view of the mitral valve of TEE again showing pseudo-prolapse of the A2 segment and restriction of the P2 segment.

At this visit, she had completed 10 days of her first BL cycle (9 mcg on days 1–7, then 28 mcg) for newly diagnosed Philadelphia-positive B-ALL, following induction with dasatinib, prednisone taper, rituximab, and intrathecal methotrexate/cytarabine. Baseline transthoracic echocardiogram (TTE) 3 months prior to initiating BL showed mild posterior leaflet thickening with trivial MR and normal biventricular function. She remained haemodynamically stable. Examination revealed a new grade 3/6 holosystolic murmur, bibasilar crackles, and 1 + ankle oedema. Laboratory tests showed creatinine 0.9 mg/dL, high-sensitivity troponin less than 6 ng/L, and NT-ProBNP 416 pg/ml. Electrocardiography showed sinus tachycardia without ischaemia. Repeat TTE and subsequent TEE revealed moderate-to-severe (3–4+) posteriorly directed eccentric MR (vena contracta 0.7 cm) due to pseudo-prolapse of the anterior leaflet from significant posterior leaflet restriction with a preserved left ventricular ejection fraction of 65% (*Figure 1*). TEE excluded vegetation, and blood cultures were negative.

Since the abrupt escalation of MR seemed unexpected, a review including a detailed, non-judgmental yet intentional history regarding prior medication use was undertaken. The patient disclosed that the ‘diet pill’ had been a 6-month course of FF taken 10 years earlier. This exposure raised suspicion for latent serotonin-mediated leaflet fibrosis, contributing to the valve’s vulnerability.

Management included holding BL therapy and starting oral diuretics with twice-daily furosemide 40 mg. We initiated GDMT with losartan 25 mg and empagliflozin 10 mg daily. Within one week, the patient's symptoms improved, and her repeat NT-ProBNP level decreased to 129 pg/ml. By week 4, her TTE showed mild MR, confirming improvement. BL was reinitiated at full dose with prophylactic low-dose diuretics (*Table 1*). The patient was followed closely by the cardio-oncology team and had office visits every 3 months. No further cardiac events occurred through her three consolidation BL cycles. Three-month and 1-year follow-up studies confirmed stable mild MR.

**Table 1:** Timeline of events

Date	Event
Aug 2023	Cryptogenic stroke, cardioembolic workup negative
Feb 2024	Diagnosed with Ph + B-ALL
Feb-Apr 2024	Dasatinib, prednisone, intrathecal MTX/cytarabine, rituximab
May 2024	Blinatumomab consolidation therapy initiated
June 2024	Dyspnoea, chest pain, severe MR diagnosed; therapy paused
June 2024	Initiation of ARBs, SGLT2 inhibitors, and diuretics
July 2024	MR severity reduced; Blinatumomab therapy resumed

## Discussion

Pivotal BL trials have not described isolated valve dysfunction. Serious cardiotoxicity is rare (<0.5%) with acute myocardial infarction, atrial arrhythmia, heart failure, and cardiac arrest each reported at an event rate of 0.4% in the Phase 3 BL trial.^1^ However, contemporary pharmacovigilance studies reveal that 20.4% of all BiTE adverse events contain more than one cardiovascular event, including bleeding, thromboembolism, hypotension, shock, and heart failure.^2^

Interleukin-6–driven CRS from BiTE therapy has been associated with diastolic impairment, which in turn may exacerbate MR by increasing preload and afterload and, through GP130–STAT3 signalling, promoting oxidative stress and mitochondrial dysfunction.^4,11^ In the presented case, CRS was notably absent, as evidenced by lack of end-organ damage and the absence of systemic inflammatory symptoms such as fever, fatigue, headache, and myalgias. Moreover, heart failure may occur independently of CRS, as demonstrated in the observational study by Sayed *et al*.^2^, in which overlap between heart failure and CRS was observed in only 17% of cases. Beyond cytokine-mediated pathways, alternative mechanisms may explain CRS-independent heart failure with blinatumomab and can potentially be extrapolated from CAR-T therapy literature.^12^ These include unmasking of subclinical diastolic impairment in individuals with pre-existing structural heart disease or direct cardiotoxicity due to cardiac antigen recognition from antigen mimicry or alloreactivity.^13^ However, published data on CRS-independent heart failure with blinatumomab remain extremely limited, underscoring the need for further mechanistic and clinical investigation.

A 1997 case series of 24 women demonstrated an association between FF–phentermine use and valvular disease.^6^ Right-sided valves were more severely affected than left-sided valves, and the histological features were identical to those of carcinoid or ergotamine-induced valve disease. Although less commonly affected, left-sided valves pose a greater haemodynamic risk. FF is a sympathomimetic amine with anorectic action mediated by the activation of brain serotonergic pathways, and phentermine interferes with the pulmonary clearance of serotonin; together, the combination potentiates circulating serotonin and produces valvular injury akin to carcinoid syndrome. Additionally, FF activates 5HT2B receptors on valvular interstitial cells, promoting glycosaminoglycan deposition and fibroelastic thickening.^6–8^ Recognition of this mechanism led to the withdrawal of fenfluramine in 1997.

The ESC cardio-oncology algorithm recommends baseline risk stratification (including history, biomarkers, electrocardiogram, and TTE) for all patients receiving BiTE and repeat echocardiography within the first treatment week when structural heart disease is present.^10^ Our patient's early re-scan enabled timely therapy escalation and prevented the progression of pulmonary oedema.

This case underscores the importance of meticulous history-taking, vigilant monitoring, and a multidisciplinary cardio-oncology approach when managing patients receiving novel immunotherapies such as BL. Our patient’s prior FF–phentermine exposure likely contributed to underlying structural mitral valve disease, which clinically manifested as elevations in left-sided filling pressures during therapy. Although CRS is the most recognized mechanism linking BL to cardiovascular events, our patient exhibited heart failure in the absence of CRS symptoms, suggesting a cytokine-independent pathway. Peer-reviewed case reports explicitly documenting heart failure without CRS are lacking; however, pharmacovigilance data indicate that cardiovascular adverse events, including heart failure, occur independently of CRS, supporting the plausibility of this presentation.^2^ Prompt initiation of GDMT, temporary cessation of BL, and careful haemodynamic optimization enabled safe re-challenge and continuation of her oncologic treatment. This case highlights that new or worsening valvular pathology during immunotherapy should not be presumed to represent irreversible drug-related cardiotoxicity. Instead, targeted evaluation and management may preserve both cardiac and oncologic outcomes. Finally, this experience reinforces the need for awareness of potential cardio-immunologic interactions, as intentional diagnostic evaluation and individualized therapy can allow patients to safely continue life-prolonging treatments. While causality cannot be definitively established in a single case report, this presentation highlights a plausible clinical association that warrants further study.

## Lead author biography


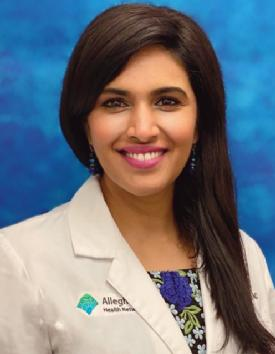
Dr. Anita Radhakrishnan, MD, is an advanced cardiac imaging cardiologist at Allegheny General Hospital, Allegheny Health Network in Pittsburgh. She specializes in cardiac MRI, cardiac CT, echocardiography, and cardio-oncology, with a focus on prevention and risk reduction. She leads community and population-health initiatives including the Every Heart Matters programme, which provides cardiovascular screening and education to underserved communities. Dr. Radhakrishnan has strong academic interests in health disparities, food insecurity, and South Asian cardiovascular risk. She is actively involved in research, medical education, and global health initiatives and is committed to improving equitable access to cardiovascular care.

## Data Availability

All data supporting the findings of this case report are contained within the manuscript. No additional datasets were generated or analysed.
